# Synergistic cellular effects including mitochondrial destabilization, autophagy and apoptosis following low-level exposure to a mixture of lipophilic persistent organic pollutants

**DOI:** 10.1038/s41598-017-04654-0

**Published:** 2017-07-05

**Authors:** Nathan E. Rainey, Ana Saric, Alexandre Leberre, Etienne Dewailly, Christian Slomianny, Guillaume Vial, Harold I. Zeliger, Patrice X. Petit

**Affiliations:** 1 Laboratoire de Toxicologie, Pharmacologie et Signalisation Cellulaire, INSERM S-1124, Université Paris-Descartes, Centre Universitaire des Saints-Pères, 45 Rue des Saints-Pères, F-75270 Paris, Cedex 06 France; 2 Laboratory for Vascular Translational Science (LVTS), INSERM U1148, X. Bichat Hospital, Université Paris 13, UFR SMBH Sorbonne Paris Cité, 75018 Paris, France; 3 Division of Molecular Medicine, Rudger Boskovic Institute, Zagreb, Croatia; 4 Laboratoire de Physiologie cellulaire, INSERM U800, Université des Sciences et Techniques de Lille 1, F-59655 Villeneuve d’Ascq, Cedex France; 5 Unité 1060 INSERM CarMen/Univ.Lyon1/INRA 1235, INSA, Bât. IMBL, La Doua 11 Avenue Jean Capelle, 69100 Villeurbanne, France; 6 Zeliger Chemical, Toxicological and Environmental Research, 41 Wildwood Drive, Cape Elizabeth, Maine, 04107 USA

## Abstract

Humans are exposed to multiple exogenous environmental pollutants. Many of these compounds are parts of mixtures that can exacerbate harmful effects of the individual mixture components. 2,3,7,8-tetrachlorodibenzo-*p*-dioxin (TCDD), is primarily produced via industrial processes including incineration and the manufacture of herbicides. Both endosulfan and TCDD are persistent organic pollutants which elicit cytotoxic effects by inducing reactive oxygen species generation. Sublethal concentrations of mixtures of TCDD and endosulfan increase oxidative stress, as well as mitochondrial homeostasis disruption, which is preceded by a calcium rise and, *in fine*, induce cell death. TCDD+Endosulfan elicit a complex signaling sequence involving reticulum endoplasmic destalilization which leads to Ca^2+^ rise, superoxide anion production, ATP drop and late NADP(H) depletion associated with a mitochondrial induced apoptosis concomitant early autophagic processes. The ROS scavenger, N-acetyl-cysteine, blocks both the mixture-induced autophagy and death. Calcium chelators act similarly and mitochondrially targeted anti-oxidants also abrogate these effects. Inhibition of the autophagic fluxes with 3-methyladenine, increases mixture-induced cell death. These findings show that subchronic doses of pollutants may act synergistically. They also reveal that the onset of autophagy might serve as a protective mechanism against ROS-triggered cytotoxic effects of a cocktail of pollutants in Caco-2 cells and increase their tumorigenicity.

## Introduction

Although oncogenetics remains a critical component of cancer biology and therapeutic research, the environmental features of tumor development and progression, such as cancer cell metabolism, have recently been reconsidered with much interest. While toxicological assessment is mandatory for every pharmaceutical drug released in global market, little is known about possible interactions caused by enviromental exposures to mixtures of pesticides. Chronic exposure to low doses of multiple pesticides, referred sometimes as a cocktail effect, is increasingly suspected of contributing to major public health issues.

In this study, we investigated the effects of mixtures of 2,3,7,8-tetrachlorodibenzo-p-dioxin (TCDD), the model of choice for studying the dioxin family, and the insecticide α-endosulfan (α-E) at sublethal dose levels. These compounds are found together in the environment as TCDD is a by-product of the endosulfan synthesis. More generally, dioxins are by-products of incineration and industrial production processes for several chloro-organic chemicals^1, 2^. These two compounds are classified by the Stockholm convention as persistent organic pollutants (POPs) due to their low degradation rates and bioaccumulation in lipohilic tissues. Both are either banned or in the process of being banned in many countries^3, 4^. Nevertheless, some countries that haven’t ratified Stockholm convention on POPs, including China, India and United States, may still use α-endosulfan in their formulations. Moreover, studies on DDT - another POP - show that, residues can be found in soil, as well as in blood and body tissues even decades after its use was discontinued. TCDD is a potent ligand of the aryl carbon receptor (AhR) which triggers multiple biological responses^5^, whereas endosulfan binds the pregnane X receptor (PXR)^6^ and the estrogen receptor alpha (ER-α). Therefore, a mixture of these two compounds presents a model for understanding the effects of multiple pathway activation.

The lipophilic characteristic of these substances exerts a key role in the initial perturbation of intracellular membranes, by providing additive effects on the linkage of the AhR receptor and to what is called the non-genomic pathway^7^.

TCDD is by far the most studied dioxin. This long lived compound is toxic and little metabolized in humans, triggering several biological responses^8^. Numerous studies have described the direct effects of this compound on mitochondria and identified the inhibition of the mitochondrial electron chain and the increased generation of ROS as one mechanism by which TCDD exerts its toxicity in the heart^9^ and liver^10–12^. TCDD treatment results in an increased production of superoxide anions released by the respiratory chain, although no alteration in mitochondrial superoxide dismutase or glutathione peroxidase is observed^11^. Interestingly, coenzyme Q levels decreased in the liver of mice after treatment, while activities of some of the mitochondrial complexes were increased^13, 14^. Low doses of TCDD also initiate oxidative stress in male germ cells that is reinforced by depletion of antioxidant enzymes. Both can alter the mitochondrial ability to supply energy^15^. These studies and others led to the proposal that TCDD causes a defect of the ATP synthase, resulting in decreased ATP^13^ as well as decreased NAD(P)H levels^16^. Production of ROS, poly(ADP-ribose) polymerase-1 activation and DNA strand breaking formation have also been reported^16^. Mitochondrial interaction with TCDD has also been reported^16–19^. TCDD has been shown to cause mitochondrial depolarization, stress signaling and tumor invasion, as well as to alter calcium homeostasis^20, 21^. Moreover, TCDD directly targets mitochondrial transcription and causes a mitochondrial phenotype which is similar to what is observed in ρ_0_ cells^20^.

Intimately related with these mitochondrial events, a decrease of the respiratory chain efficiency and a defect in ATP synthase activity are known to cause a decrease in ATP production, suggesting a TCDD-induced apoptosis. A huge controversy has arisen on this subject, since multiple authors agree with a TCDD induction of apoptosis^22–26^, while others argue that an inhibition/delay of apoptosis is at hand^27–29^. A few researchers have started investigating TCDD-induced autophagic processes^30, 31^. We believe that recent work provides clues to reconcile all these different views. Since autophagy may mask the reality of mitochondrial apoptosis induced by TCDD and may play an essential role in the increased susceptibility to tumorigenesis, we believe that the cross-link between autophagy and cell death is the primary point to be investigated.

Like TCDD, endosulfan generates reactive oxygen species with contradictory assertion regarding apoptosis. Despite data describing ΔΨm drop, cytochrome *c* release and mRNA levels inhibition leading to the activation of caspase-3 and apoptosis^32^, it appears that the initial signaling is clearly related to autophagic processes and therefore, has carcinogenic effects^33^.

When treated with mixtures of pollutants, sperm motility was significantly decreased in animals that received a mixture of dieldrin, endosulfan, dicofol, dichlorvos, and permethrin^34^. Taken together, these results indicate that endosulfan profoundly alters the phenotype of liver cells by inducing cell detachment and partial epithelial-mesenchymal transition as well as disrupting the anoikis process^35^. All these events account, at least in part, for the carcinogenic potential of endosulfan in the liver.

Here, we hypothesize that the initial effects of both α-E and TCDD could be related primarily to the interaction of these lipophilic substances with subcellular compartments, as the toxicity of chemical mixtures composed of multiple lipophilic species has been associated with total lipophilic load^36–38^. The endoplasmic reticulum or the lysosomes are examples of such subcellular compartments, since oxidative stress^39–42^, lipid peroxidation^43^ and calcium rise^44, 45^ have been shown to be associated with endosulfan treatments and can be associated with cell death induction^41, 46^ or more precisely with apoptosis^47–49^. It also seems that these two pollutants act in a general inflammatory context. Effectively, the inflammatory capabilities of TCDD have been highlighted within hepatocytes, where TCDD elicits dose-dependent hepatotoxicity including fat accumulation, inflammation, and fibrosis which may progress to hepatocellular carcinoma^50^. TCDD acts via the increased transcriptional activities of CYP1A1 (Cytochrome P450, family 1, subfamily A, polypeptide 1) and inflammatory cytokines^51^. Similarly, recent data argue that exposure to α-E increases the secretion interleukin-6 and -8, suggesting its involvement in inflammation^32^.

The objective of the present study was to investigate the combined effects of mixtures of pollutants used at sub-lethal doses, taking into consideration that these compounds may have very early effects on diverse cellular membrane systems due to their lipophilicity. Here we reconcile data from various sources, especially that of the role of a mixture of TCDD and α-Endosulfan, on calcium rise, early mitochondrial events, autophagic processes and apoptosis.

## Results

### Subtle effects of TCDD and Endosulfan on plasma membrane and cell death

**Figure 1:**
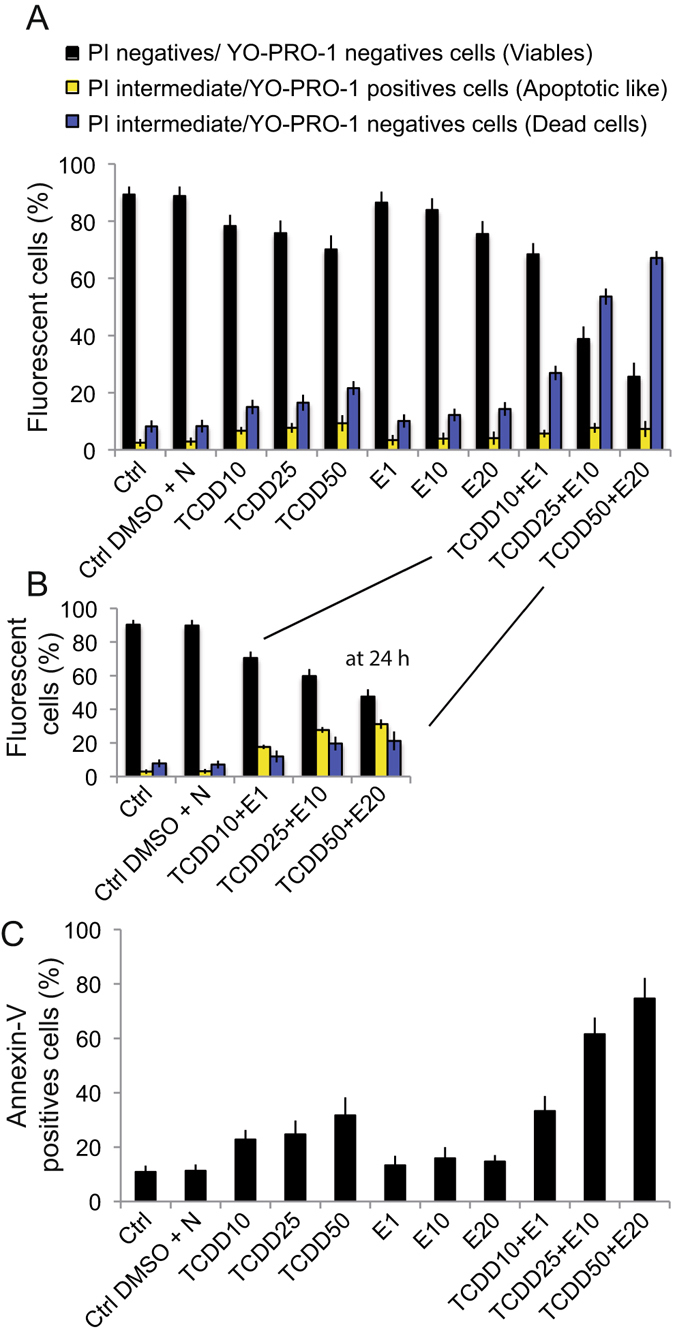
TCDD plus endosulfan induces cell death. (**A**) YO-PRO-1/PI staining of Caco-2 cells treated with TCDD or α-E alone or with TCDD + endosulfan mixtures for 48 h incubation. Two controls have been used: control cells (in DMEM) and control cells treated with DMSO and nonane since TCDD and E are dissolved respectively in nonane or DMSO. Each experiment has been performed each time we set flow cytometry analysis of any other parameters (ΔΨm, ROS, annexine V/PI measurements or caspases, cathepsin or calpain determination). So the number of experiments realized is high (n ≥ 55). (**B**) YO-PRO-1/PI staining of Caco-2 cells treated with increased amounts of TCDD + endosulfan mixtures for 24 h incubation. (**C**) Phosphatidyl serine exposure to the outer leaflet of the plasma membrane measured with annexine V-FITC with Propidium iodide (n = 12).

A primary assertion made here is that both TCDD and α-E could interfere with the plasma membrane and then modify the cellular viability. It is for that reason that we investigated the cellular viability (plasma membrane permeability) using the YO-PRO-1 and propidium iodide assay designed for flow cytometry (Fig. 1A). When challenged with YO-PRO-1/PI, the cells treated 48 h with the TCDD + E cocktails (TCDD 10 nM + α-E 1μM, TCDD 25 nM + α-E 10 μM and TCDD 50 nM + α-E 20 μM) provided us with curious results at 48 h, as we were unable to detect a consequent subpopulation of YO-PRO-1^+^ cells with PI^intermediate^ fluorescence, which usually corresponds with apoptotic cells. Moreover, it appeared as a singular population in which YO-PRO-1^−^ and PI^intermediate^ is different from viable cells (YO-PRO-1^−^/PI^−^) and also from the classical population designed as dead cells (YO-PRO-1^+^/PI^+^). This population is clearly one of permeabilized cells which has lost the YOPRO-1 fluorescence and a part of the PI fluorescence. This corresponds to being in a late necrotic state. Due to that, we quantified the viable cells, the dead cells after apoptosis and this necrotic population (Fig. 1A). After a 24 h treatment with the cocktails, the transient apoptotic population is visible (Fig. 1B) but the quantity of dead cells remains quite low. The differences in cellular permeability of the cells treated by the two products separately or together (Fig. 1A) highlight the fact that TCDD appeared to be a very effective inducer of the loss of cell viability. The cocktail is much more effective even if endosulfan alone was nearly inefficient at the concentrations used.

The use of a complementary test using the usual annexin-V/PI flow cytometric analysis confirms the results obtained with YO-PRO-1/PI staining. Therefore, compared with the controls, TCDD alone (at 10, 25 and 50 nM) does not induce a very significant amount of cell death (no more than 30%) and neither does the endosulfan alone (Fig. 1B) whereas the association of the two products acts synergistically to provoke elevated levels of death (up to 60%at TCDD 25 + E 10 and as high as 75% of cells for TCDD 50 + E 20 for 48 h incubation). Accordingly, it seems that TCDD and α-E mixtures deeply change the plasma membrane permeability of the cells exposed to such treatments. These plasma membrane changes are also evidenced by the positive response to Annexine V that can be ascribed to TCDD addition but is mostly due to the effects of mixtures of pollutants (Fig. 1C).

We tested β-hexaminidase release into the cellular environment since it has been reported that TCDD or the cocktails of pollutants (TCDD + α-E) affect not only the cellular permeability but also the exocytosis^52^. This can be seen in supplemental Fig. 1 where the efficiency of the cocktails of pollutants to enhance the β-hexaminidase exocytosis could be compared with the slight effect of TCDD alone.

### TCDD and α-E increase lipid peroxidation, protein carbonylation, lactate release and EROD activity, in a concentration dependent manner

**Figure 2:**
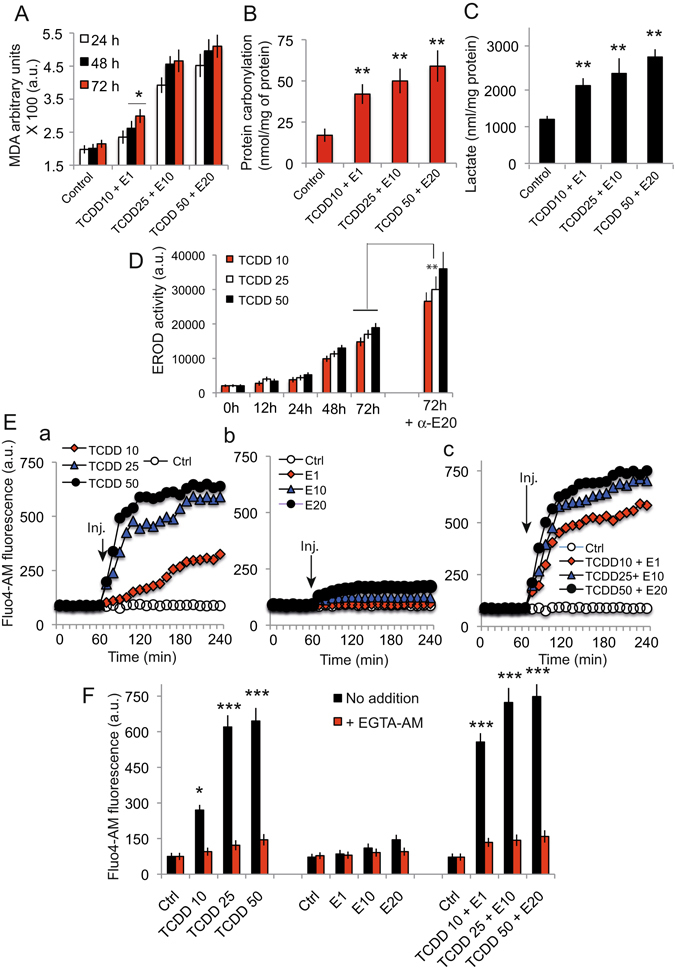
Diverses cellular activities linked to the pollutant cocktail treatment. (**A**) Malondialdehyde production as a function of time (24 h, 48 h and 72 h) and TCDD + E concentration (n = 5). (**B**) Protein carbonylation in cells treated with the indicated concentrations of pollutant cocktails, after 48 h incubation (n = 6). (**C**) Lactate production induced by the treatment of Caco-2 cells after 48 h treatment with different pollutant cocktails. The data are expressed as nmol/mg protein (n = 7). (**D**) EROD activity linked to TCDD treatment at different concentration and an example of the synergistic action of TCDD + α-E at 48 h incubation (n = 5). (**E**) Calcium rise induced by the individual pollutants (i.e, TCDD or endosulfan) or the pollutant mixtures at different concentrations in presence or absence of EGTA-AM during the 4 hours following exposure (n = 7). (**F**) Histograms of the calcium content of the cells after treatments (similar to E) but at 48 h in presence or absence of EGTA-AM (n = 7).

To examine the many damaging effects induced by the pollutant mixtures - suggested by the loss of plasma membrane permeability, exposure of phosphatidyl serine moeties outside of the plasma membrane and β-hexaminidase release- we first studied the lipid peroxidation that was tested by measuring the malondialdehyde (MDA) produced in Caco-2 cells. The production of MDA was significantly increased with increasing cocktail concentrations, compared to the DMSO + nonane-treated control cells (Fig. 2A). These results suggest that the different mixtures increase the production of peroxidized lipids in a concentration-dependent manner even with a sub-lethal combination of cocktail (i.e. TCDD 10 nM and α-E 1 μM). Such an apparition of peroxidized lipids is usually linked to a level of oxidative stress that may drive the onset of autophagy, mitophagy, and aerobic glycolysis resulting in the local production of high-energy mitochondrial fuels (such as L-lactate, ketone bodies, and glutamine). Next we investigated protein carbonylation (Fig. 2B) and L-Lactate production (Fig. 2C). Clearly, there was a protein carbonylation increase in cells exposed to the cocktails (Fig. 2B). The stress increase in exposed cells is also associated with the release of more lactate in the cellular environment (Fig. 2C). The induction of the cytochrome P450 monooxygenase 1 A (CYP1A) as shown by 7-ethoxy-resorufin-O-deethylase (EROD) activity after treatment with different mixtures of TCDD + α-E (Fig. 2D) could also been related to all these events. Three different mixtures - TCCD 10 + α-E 1, TCDD 25 + α-E 10, TCDD 50 + α-E 20 - all induce an EROD activity which is time and concentration dependent. There is clearly a synergy when α-E is added to TCDD (72 h time point, Fig. 2D).

### TCDD and α-E treatment induce a calcium rise

Since TCDD has previously been described to increase intracellular Ca^2+^ levels significantly^52–54^, we looked at TCDD-mediated Ca^2+^ rise and its ability to trigger β-hexaminidase exocytosis in Caco_2_ cells.

The measurement of intracellular calcium with Fluo-4-AM (*Figures E a-c and F*) suggests that TCDD is the main inducer of ER calcium homeostasis disruption whereas endosulfan has almost no impact. However, the cocktail of pollutants TCDD + α-E at all concentrations tested appeared to act synergistically and increase the calcium level in the cytoplasm. These results show that cocktails of pollutants, even used at sublethal doses, induce a calcium rise resulting from the destabilization of the endoplasmic reticulum. We found that the calcium rise could be totally prevented with EGTA-AM (Ethyleneglycol-bis(β-aminoethyl)-N,N,N′,N′-tetraacetoxymethyl Ester), an efficient cell penetrating calcium chelator (Fig. 2F).

### TCDD treatment disrupts ER ultrastructure of Caco-2 cells and the mixture of TCDD + α-E potentiates ER swelling and damage

An alteration of calcium homeostasis seems to be a common thread for TCDD toxicity^52^. Since, Caco_2_ cells do not have T-type channels, the increase of cytosolic calcium could not be related to the plasma membrane destabilization that was observed. We hypothetized that the calcium rise precedently recorded in the presence of TCDD or the pollutant cocktails might originate from the endoplasmic reticulum.

**Figure 3:**
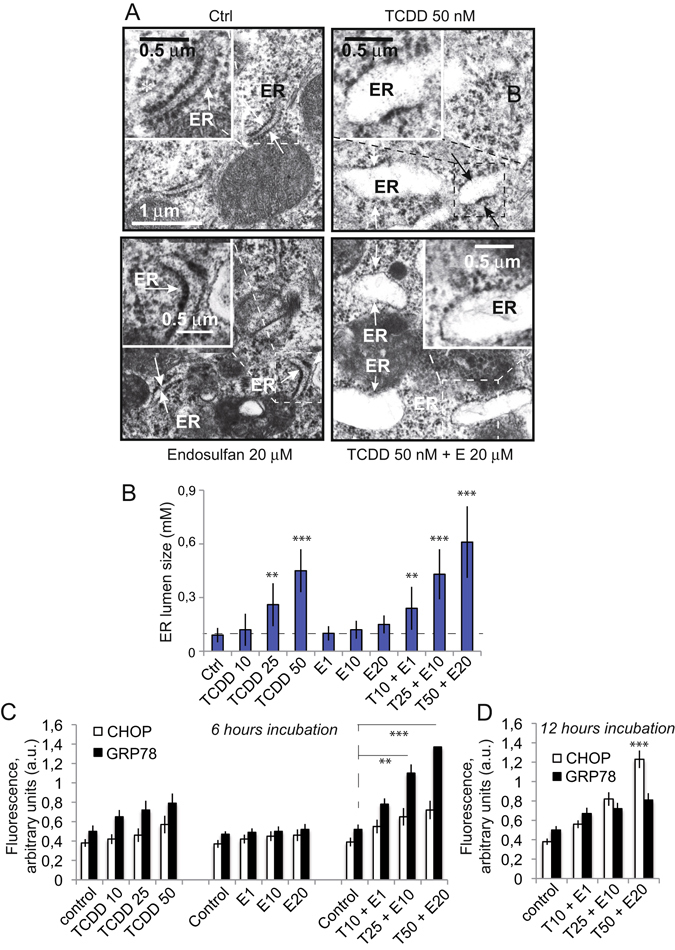
TCDD + endosulfan induced ultrastructural ER alterations in Caco-2 cells. (**A**) Caco-2 cells were treated with or without different substances separately or in cocktails (i.e., control; endosulfan 20 µM; TCDD 50 nM and TCDD 50 nM + Endosulfan 20 µM) for 24 h and then examined by transmission electron microscopy. Representative images from five independent experiments are shown. ER, endoplasmic reticulum; M, mitochondria; N, nucleus; NmL nuclear membrane lumen; SF, stress fibers. Arrows indicate normal or dilated ER. *P < 0.05 compared with the control group. Representative images from five independent experiments are shown. (**B**) Histogram presentation of the quantification of the width of the ER lumen (in µm) in Caco-2 cells treated with either TCDD 10, 25 or 50 nM; endosulfan 1, 10 or 20 μM or a mixture of TCDD + Endosulfan (respectively 10 nM + 1 μM; 25 nM + 10 μM or 50 nM + 20 μM). Data are shown as mean ± SD (n = 11). (**C** and **D**) Flow cytometric determination of the CHOP and GRP78 activities linked to ER stress induced by TCDD, α-E and TCDD + α-E treatements. (**C**) After 6 h treatment and (**D**) after 12 h treatment for the mixture only.

We evaluated the effects of TCDD and endosulfan on endoplasmic reticulum (ER) morphology using transmission electron microscopy. The ER ultrastructure of control Caco-2 cells had a normal flattened appearance (Fig. 3A). ER dilation was measured as the width of the lumen in each Caco-2 sample. TCDD 50 nM treatment for 48 h resulted in obvious ER dilation in the Caco-2 cells examined whereas a 20 μM endosulfan treatment had almost no effect (Fig. 3A,B). DMSO + nonane controls or treatments with 1 nM TCDD had no significant effect on ER ultrastructure, with a lumen width of respectively 0,10 ± 0,03 µm and 0,13 ± 0,04 µm (not shown). However, 10, 25 or 50 nM TCDD resulted in obvious ER dilation (Fig. 3A), and the widths of the ER lumen in cells treated with 10, 25 and 50 nM TCDD were significantly higher (0,17 ± 0,011; 0.25 ± 0.012 µm and 0.44 ± 0.012 µm respectively) compared with controls (0.10 ± 0.02 µm). Cocktails of TCDD and endosulfan clearly potentiated the increase of ER lumen width even at concentrations where compounds isolated are ineffective (TCDD 10 nM and α-E 1 μM for example) (Fig. 3A,B).

### Consequences of ER stress as measured by CHOP and GRP38 activation

In accordance with the ER stress pathway revealed by the ER swelling on electron photomicrographs (Fig. 3A), we found that TCDD slightly induced the expression of GRP78 (78-kDa glucose-regulated protein (GRP78, also referred as BiP or HSPA5) and CHOP (CCAAT-enhancer-binding protein homologous protein), two markers of ER stress, in a concentration-and time-dependent manner (Fig. 3C,D). It should be noted that treatment of cells with 1 nM or 10 nM TCDD for 6 h, did not significantly induce CHOP expression (Fig. 3C) but exacerbated GPR78 expression in these treated cells. However, when Caco-2 cells were exposed 12 h to TCDD, GRP78 expression is peaked at 6 h after treatment (Fig. 3D). In contrast, the highest increase in CHOP expression was observed after 12 h of treatment. In comparison, α-E alone (1–20 μM) does not seem to affect CHOP and GPR78, nevertheless, TCDD and α-E act in a synergistic way since both GPR78 and CHOP activities were significantly increased when cells were treated with any concentration of the cocktail of pollutants. These results are also in line with ER stress induced by other pollutant cocktails^55, 56^ and are also consistent with previous findings^23, 57^ in which higher concentration of TCDD alone (i.e. 100 nM) was described as inducing a canonical ER stress.

### TCDD + Endosulfan mixture affects mitochondrial structure and function

**Figure 4:**
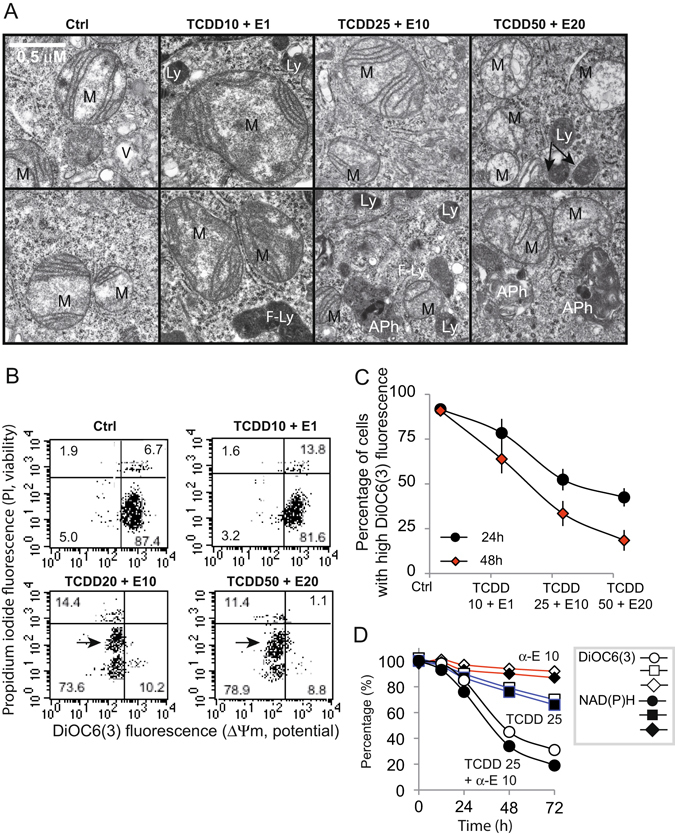
Mitochondria behavior of TCDD + α-E treated cells. (**A**) Electron microscopy pictures of Caco-2 cells treated 48 h with different TCDD + α-E cocktails. Two pictures for each treatment are presented as representative of the subcellular organelles affected by each treatment (top and bottom). M; Mitochondria, Ly; lysosomes, APh; autophagosomes, F-Ly; Fused lysosomes. The bar in the first top left panel represents 0.5 μM. (**B**) Biparametric flow cytometric analyses of the mitochondrial membrane potential [ΔΨm, DiOC_6_(3)] and permeability of the plasma membrane to propidium iodide (PI). The arrow indicates a cell subpopulation (SP) with low ΔΨm and intermediate staining for PI. (**C**) Evolution of the mitochondrial membrane potential (ΔΨm) as measured by DiOC_6_(3) staining of cells treated 24 h or 48 h with different TCDD + α-E cocktail (n- 18). (**D**) Evolution of the mitochondrial membrane potential [ΔΨm, DiOC_6_(3)] and of the NAD(P)H cellular content as a function of time for an incubation with a TCDD25 + α-E 10 cocktail, TCDD25 and a-E alone (n = 20, variations are low and not visibles due to the size of the points used for drawing).

The observation of control and treated-cells by electron microscopy demonstrates an interesting mitochondrial behavior following exposure to the increased concentration of the mixture cocktail (after 48 h treatment) (Fig. 4A). Mitochondria of mainly glycolytic control cells look roundly shaped, with a double membrane and few but well organized cristae that are transverse into the matrix (upper panels in Fig. 4A). Vacuoles (V) and normal ER were also observed. In TCDD10 + α-E1 treated cells, the mitochondria have a swollen matrix and some of the cristae membranes seem to be less organized. Lysosomes (Ly) or even possible fused-lysosomes (F-Ly) were observed in these treated cells. An increased concentration of TCDD and α-E (TCDD 25 + α-E10) showed the mitochondria of treated cells to be smaller, with less organized cristae membranes, and surrounded by a huge quantity of lysosomes [individual (Ly) or fused (F-Ly)] and even likely by autophagolysosomes (APh). At the highest TCDD + α-E concentration (TCDD 50 + α-E10), the mitochondria are numerous, even smaller and show a loss of most of their cristae membranes. Their matrix appears to be translucent. Lysosomes and many autophagic vesicles (autophagosomes, A-Ph) were also detected on the images (Fig. 4A).

It, therefore, seems that the mitochondria undergo a clear swelling at low mixture concentration and evolve slowly towards a more fragmented and translucent appearance when the cocktail concentrations increase. One might believe that these mitochondria are indeed fragmented (they undergo fission) and representative of mitochondrial bioenergetic failure.

Very often cell death processes are more or less linked to mitochondrial membrane potential (ΔΨm) impairments and disruption of the mitochondrial homeostasis^58, 59^. Accordingly, we decided to analyze the mitochondrial membrane potential (ΔΨm) by flow cytometry with DiOC_6_(3) [lipophilic dye 3,3′-dihexyloxacarbocyanine Iodide] (Fig. 4B–D). Biparametric histograms [DiOC_6_(3) versus PI)] show a drop of ΔΨm when cells are treated with a mixture of TCDD and α-E at high doses. The treatment of cells with a mixture of TCDD + α-E induces a specific drop in ΔΨm which is time and concentration dependent (Fig. 4B,C). In Fig. 4D, it is clearly shown that the drop ΔΨm paralleled a drop in NAD(P)H fluorescence when cells were treated with the cocktail of pollutants at the medium concentration, i.e. TCDD 25 nM + α-E 10 μM, whereas α-E treatment alone had almost no effect on the ΔΨm and TCDD alone only a slight effect on both ΔΨm and NAD(P)H fluorescence.

### Mixtures of pollutants affect the cellular bioenergetics

**Figure 5:**
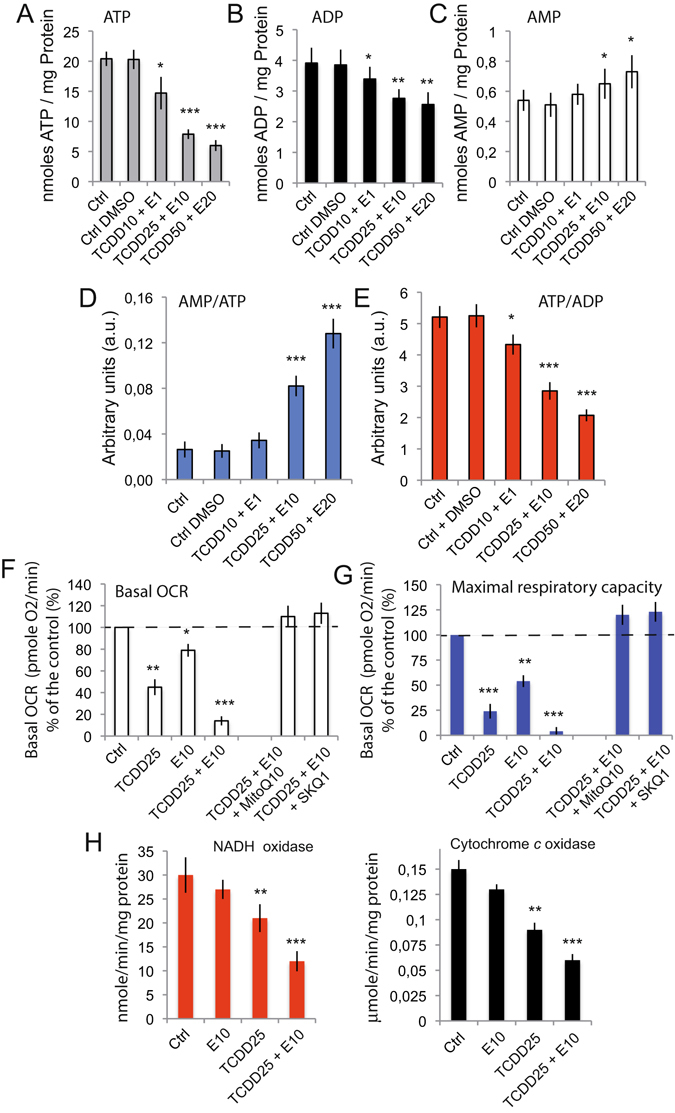
Analysis of diverses bioenergetic parameters concerning cells or mitochondrial isolated from cells which have been treated by diverses TCDD + α-E cocktails. (**A**–**E**) HPLC determination of the ATP, ADP and AMP levels in cells treated with different concentrations of TCDD + E (dosage expressed as nmoles/mg protein). AMP/ATP and ATP/ADP ratios are respectively presented in D and E (arbitrary units). From A to E, the statistics are based on 6 differents experiments (n = 6). (**F** and **G**) Seahorse analysis of the cellular respiration for Caco-2 cells treated with TCDD or α-E, or TCDD + α-E cocktails. (**F**) basal oxidative respiratory chain (ORC) and in (**G**) Maximal respiratory capacity after 10 μM mClCCCP addition (n = 4). On these two panels F and G, the mitochondrially targeted antioxidants MitoQ10 100 μM and SKQ1 5 nM have been used to minimize the superoxide anion production. TCDD alone at 25 nM, α-E at 10 nM and the cocktail TCDD 25 nM + α-E 10 nM. MitoQ10 is used at 500 mM and SKQ1 at 25 μM. (**H** and **I**) NADH oxidation (**H**) and Cytochrome *c* oxidase activity (**I**) of isolated mitochondia treated with α-E 10, TCDD 25 or a mixture of TCDD + α-E (25 + 10) (n = 8).

AMP, ADP and ATP (adenosine monophosphate; ADP, adenosine diphosphate and Adenosine triphosphate, respectively) measurements showed an ATP and ADP drop in cells treated with the different mixtures of pollutants (Fig. 5A–C). As a consequence, the cellular lysates issued from of Caco-2 cells treated with the different pollutant cocktails exhibited an ATP/ADP ratio lower than for the control cells (Fig. 5E). In theory, [AMP] is proportional to [ADP]^2^/[ATP] (assuming a close equilibrium with adenylate kinase in most cells). Thus, if the absolute ATP and ADP levels are taken into account together with [AMP], it is clear that [AMP] is increased by the different treatments. This finding is important, because an increase in AMP/ATP ratio (from 0,021 ± 0,004 in control cells to almost 0,095 ± 0,008 in cells treated with TCDD50 + α-E20) (Fig. 5D) might lead to the activation of the AMP kinase, a major regulator of the mitochondrial biogenesis via the PGC1-α pathway, thus accounting for the observed increase in mitochondrial mass (even if represented by smaller mitochondria).

When the whole cell respiration is recorded by Seahorse measurement it is obvious that the basal oxygen consumption rate (OCR) and maximal respiratory capacity (MRC) are affected to different extents by α-E and TCDD and also, that pollutant mixtures demonstrated a synergistic inhibition of the basal OCR and MRC (Fig. 5F,G). Also surprisingly is the fact that mitochondrially targeted antioxidants like MitoQ10 and SKQ1 are able to fully restore both OCR and MRC (Fig. 5F,G), and, in addition, protect the mitochondria from basal ROS level production usually associated with the normal function of the respiratory chain due to the OCR and MRC being slightly higher than the controls).

### TCDD plus endosulfan treatment initiates cellular light scattering changes associated with ROS production

**Figure 6:**
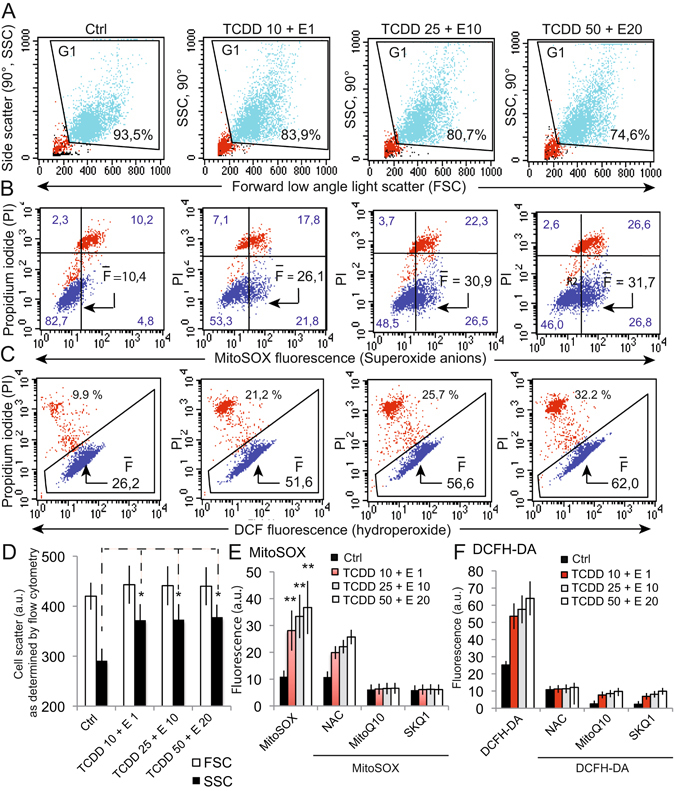
Light scattering properties and ROS production of Caco-2 cells treated with different concentrations of the pollutant mixture TCDD + E. (**A**) Changes in light scattering (i.e forward low angle light squatter, FSC and side squatter, SSC) of Caco-2 cells treated 48 h with different mixtures of TCDD + E. Percentages (%) indicate the amount of cells selected on FSC/SCC that are not debris (red dots at the bottom left part of the histograms) or aggregates. (**B**) Biparametric analyses of cells selected in the gate G1 on the light scatter (**A**), for the MitoSOX fluorescence (superoxide anion production) versus propidium iodide (PI) staining (taken as plasma membrane permeability and/or cellular viability). F bar (mean fluorescence value) indicates the mean fluorescence of the population in blue (viable cells) whereas the other values (4) are the percentage of cells in each quadrant. Red dots represent PI positive non-viable cells. (**C**) Biparametric analyses of the cells selected in the gate G1 on the light scatter (**A**), for the _2_HDCF-DA staining and the DCF fluorescence (H_2_O_2_ production, in blue) versus propidium iodide (PI, in red) staining (taken as plasma membrane permeability and/or cellular viability). F bar indicates the mean fluorescence of the population in blue that increases upon treatments. The percentage of cells that are non-viables (PI positives) is indicated in % (red dots). (**D**–**F**) The histograms represent statistical analyses of the above A, B and C measurements respectively concerning in (**D**) the light scattering properties (*P < 0,5), in (**E**) the superoxide anion generation with MitoSOX staining and in (**F**) the hydroperoxide measurements with DCFH-DA staining. Means of 7 independent experiments are presented (n = 7). In (**E**) and (**F**) respectively, MitoSOX and DCFH-DA measurements have also been realized in presence of three different antoxidants, i.e., N-acetylcystein (NAC) as a cytoplasmic anti-oxidant and two mitochondrially targeted antixoxidants which are MitoQ10 and SKQ1. (all data are means of 6 independent experiments, n = 6).

Flow cytometric analyses of the light scattering properties of control cells and cells treated with increasing concentrations of the pollutant cocktail showed an increase in the light scattering properties of treated cells (Fig. 6A,B). Indeed, the number of cells in gate 1 (G1) combining forward light scatter (FSC) and side scatter (SSC, 90° angle) slightly decreased when pollutant cocktail doses increased (suggesting « cell death »). However, the main characteristic of these cells is an important increase in SSC without any significant change in FSC (Fig. 6A,D). Moreover, these light scattering changes could be associated with an increased production of superoxide anions (Fig. 6B,E). Since 0^2•−^ do not diffuse far from their site of production, mainly mitochondria, the dead cells appeared also to be positive for 0^2•−^. We observed as well an increased production of H_2_O_2_ in cells treated with the mixture of pollutants (Fig. 6C,F). In contrast to 0^2•^, H_2_O_2_ is more diffusible. This may explain why dead cells that are permeable do not retain their staining and exhibit a lower fluorescence than viable cells. These experiments clearly suggest that TCDD + α-E treatment induces the swelling of cells, a fact that is usually associated with autophagy.

### TCDD and endosulfan induces lysosome proliferation and fusion

**Figure 7:**
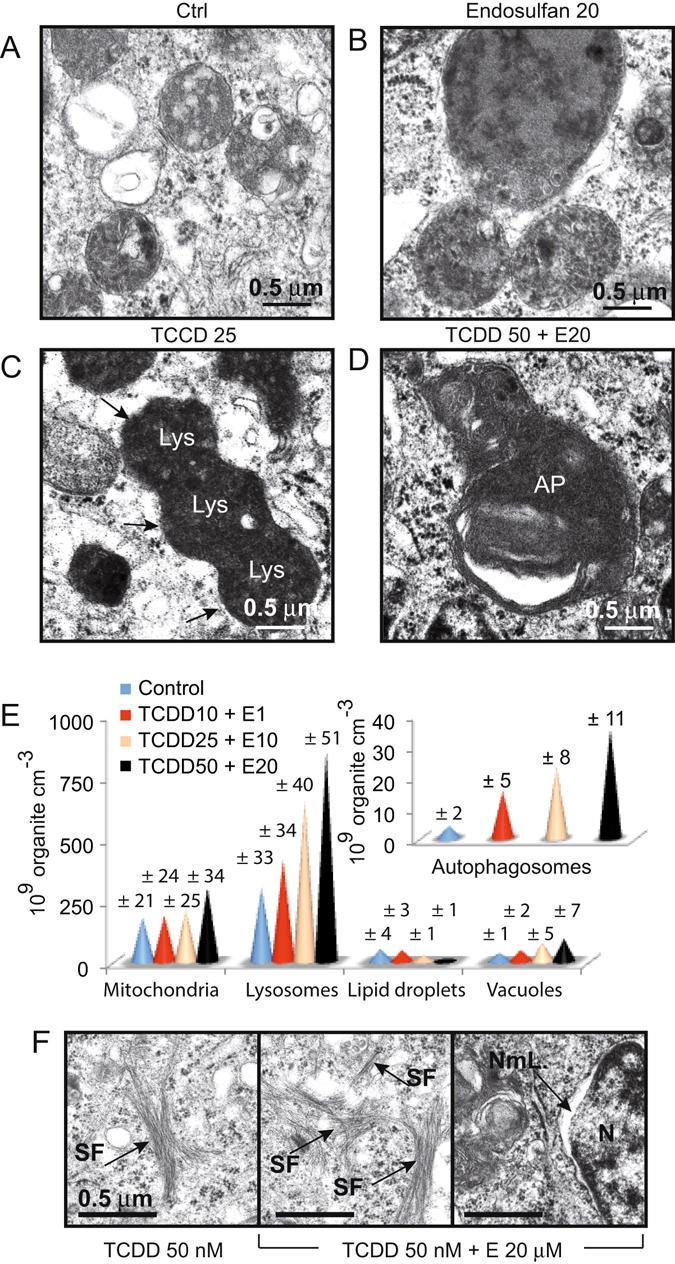
Morphology of the lysosomes and details of the cytoplasm in cells treated with a cocktail of polluants. (**A**–**D**) Caco-2 cells were treated for 24 h with 20 µM endosulfan, 50 nM TCDD or 50 nM TCDD combined with 20 μM endosulfan and observed under transmission electron microscopy. Typical lysosomes are shown in control (**A**) When cells are treated with α-E (20 μM) alone, the lysosomes appeared swollen (**B**) whereas with TCDD alone (50 nm) the lysomes become electron dense and seem to fuse to each other (**C**). When the sample has been treated with a cocktail of pollutant, i.e., TCDD 50 nM and α-E (20 μM), autophagosomes are visible (**D**,**E**) Relative aboundance of each subcellular compartment estimated from electron microscopy picture and counted. The mitochondria, lysosomes, lipid droplets and vacuoles are counted on one side and the autophagosomes upper left pannel are counted apart. Statistical analysis calculated from 10 separated experiments and from 5 differents microscopic field (pooled) for each (n = 10). (**F**) Electron microscopy picture of Caco-2 cells treated with 50 nM TCDD alone or a cocktail of TCDD 50 nM + 20 μM Endosulfan. SF: stress fibers and NmL, nuclear membrane lumen.

The increasing number of lysosomes associated with the changes in mitochondrial structures after TCDD and endosulfan treatment prompted us to analyze lysosomal behavior (Fig. 7). Lysosomes of control cells look normal and are in the vicinity of either vacuoles (Fig. 7A) or mitochondria. Treatment with 20 μM α-E, clearly enhanced the number and the size of lysosomes within a single cell (Fig. 7B), whereas treatment with 25 nM of TCDD seemed to be sufficient to induce lysosomal fusion within the cytoplasm (Fig. 7C). The cocktail of pollutants (TCDD 50 + α-E 10) provokes a massive increase of autophagolysosomes in treated cells (Fig. 7D). Overall, we found that mitochondria first swell and slightly increase their number before becoming smaller (fission) with empty matrices and lacking cristae membrane in cells treated with increasing doses of pollutant cocktails (Fig. 4A). Similarly, the lysosome compartment was deeply modified by the mixture of TCDD and α-E, as lysosomes drastically increased in number in cells treated with increasing concentrations of pollutant cocktails. We, however, observed a decrease in lipid droplet numbers, and an increasing number of autophagosomes after treatment with increasing doses of TCDD + α-E (Fig. 7E).

We find it remarkable that treatment with TCDD, but not with endosulfan, induces stress fiber apparition, (a situation that is even more pronounced with the TCCD 50 + α-E 20 treatment), together with lumen apparition at the nuclear membrane level (Fig. 7F). It should be noted that the electron micrographs also reveal stress fibers in the cytoplasm and showed a lumen structure at the nuclear membrane of cells treated with the TCDD 50 + α-E 20 cocktail (Fig. 7F).

### Mixture of pollutants induces autophagy and cell death

**Figure 8:**
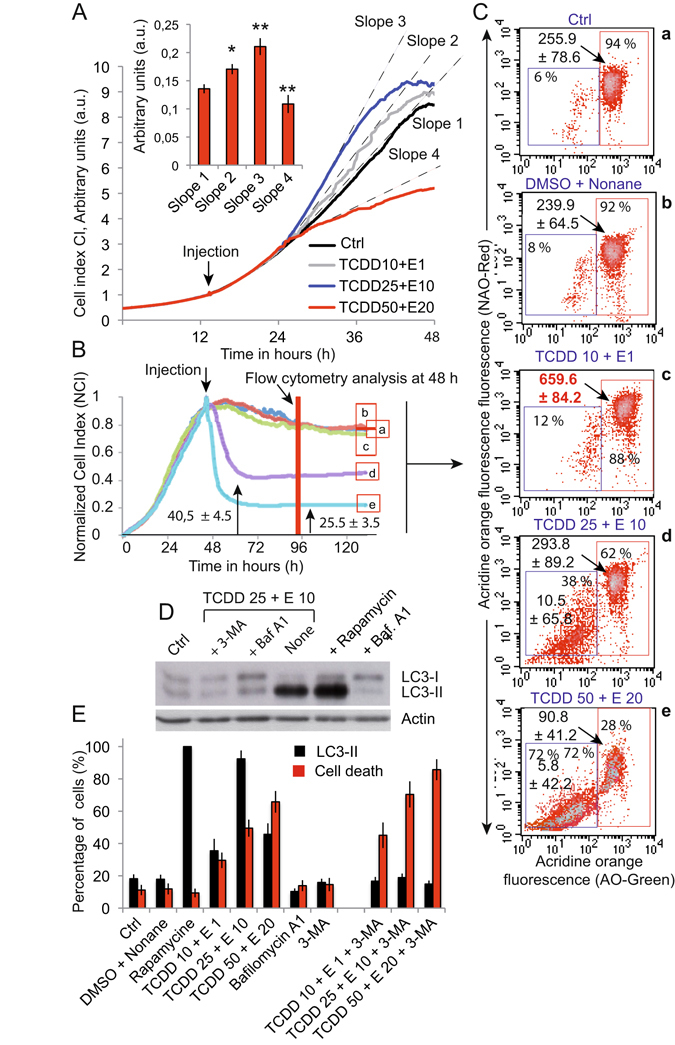
Measurements of Caco-2 cellular proliferation changes upon treatment with different mixtures of TCDD + E (xCELLigence) and of the induction of autophay associated processes. (**A**) xCELLigence measurements of Caco-2 cell proliferation in presence or absence of different mixtures of TCDD + E. Treatments were realized at the early time of the proliferation curve and the slope of the impedance curve is measured as an index of cellular proliferation. An additional histogram resume measurements on a statistical basis (n = 6). (**B**) xCELLigence measurements of Caco-2 cell proliferation realized by exposure to a mixture of pollutants at the late stage of the proliferation curve (80% of the maximum). Cells were untreated (curve a) or treated with DMSO plus nonane (curve b), TCDD 10 + E 1 (curve c), TCDD 25 + E 10 (curve d) or TCDD 50 + E 20 (curve e). Flow cytometric analysis indicates the time at which cells were extracted from XCELLigence device for analysis with indications for different treatments. (**C**) Analysis of the intracellular acidic compartment by acridine orange staining for the cells issued from the xCELLigence device (a to e). Note that the red fluorescence indicates the importance of the acidic compartment within the cell. A decrease in green fluorescence is linked to an increased number of dead cells. Red fluorescence decreases also since the dismantling of the cell abolishes the difference in pH between the cytoplasm and the altered acidic vesicles (whatever they are lysosomes or autophagic vesicles). (**D**) Western blots of LC3-I and LC3-II of control and treated cells (actin serve as a reference). Cells left untreated or exposed to the pollutants were analysed in presence of absence of Rapamycin 100 nM (Rapamycin is also an inducer of autophagy, as inhibition of mTOR mimics cellular starvation by blocking signals required for cell growth and proliferation) or of 3-MA and of 1 μM Bafilomycin A1 (an inhibitor of the fusion of autopagosomes to lysosomes). (**E**) LC3-II fluorescence (in red) and cell death (taken as the cell population with a ΔΨm drop) in black squares (n = 5 for each mesurement).

Using a cellular impedance measurement device (RTCA, ACEA, Invitrogen), we measured the Caco-2 cell proliferation in untreated and pollutant cocktail treated conditions (Fig. 8A,B). When added early in the proliferation phase, the lowest cocktail concentrations (i.e. TCCD 10 + α-E 1 and TCDD25 + α-E 10) slightly exacerbated cellular proliferation, as depicted by the changes in the cell index, compared with the control (Fig. 8A). At higher concentration of TCDD 50 + α-E 20 a drastic inhibition of the cellular proliferation was observed (Fig. 8A, *slope 4*). We have previously seen that cells treated with TCDD + α-E pollutant cocktails swell (higher side scatter than control cells) (Fig. 4A). Cell size upon expansion on the electrode is one of the main components of the Cell Index measurement (CI). Therefore, the increasing CI observed in Fig. 8A, could not only be related to an increase in the proliferation of the treated cells but also to cellular swelling. With the two first treatments (i.e, TCDD 10 + α-E 1 and TCDD 25 + α-E 10) the initiation of autophagy may lead the cells to swell, indeed mimicking an increased cellular proliferation (Increased CI). At higher TCDD + α-E concentration (TCDD 50 + α-E 20) cellular proliferation is inhibited and the CI decreased (Fig. 8A). It is hypothesized that in these conditions cells first swell, since there is an induction of autophagy after treatment.

When the pollutant cocktails were added at the proliferation phase, we observed that the addition of TCDD10 + α-E1 gave rise to a CI curve almost similar to that for untreated cells or cells treated with DMSO + Nonane (Fig. 8B *a*–*c*), whereas treatments with higher concentrations of TCDD + α-E (Fig. 8B *d and e*) induced a drop of CI likely directly linked to the death of the treated cells (Fig. 8D). Once again, the question of protection from death by early autophagic events was raised. To answer this question, we analyzed the variation of the intracellular acidic compartment of the cells treated in a similar manner (Fig. 8C) as electron microscopy images showed a huge increase in lysosomes and in autophagosomes in treated cells as the OA red staining is increased (Fig. 8C *a*–*e*). Acridine Orange (AO) effectively simultaneously stains the DNA/RNA molecules in green (AO-Green) and the acidic compartment in red (AO-Red). We observed a decrease in the green fluorescence when cells were treated with increasing concentrations of the cocktail of pollutants, which is characteristic of cell death, as cell death processes are usually accompanied by DNA compaction (lower accessibility of the DNA for AO intercalation). An significant increase of the AO red florescence could be observed when cells were treated with TCDD 10 + α-E1, which suggests an increase in the acidic compartment likely due to increased autophagic vesicles and their fusion with the lysosomes as well as an increase in lysosome number as previously described (Fig. 7E). With higher concentrations of TCDD + α-E (≥TCDD 25 + α-E10 and of course TCDD 50 + α-E20), a new sub-population of cells appeared. This sub-population of cells was characterized by low AO-Green and lower AO-Red fluorescence, suggesting alterations of the acidic compartment, in the context of significant cell death (respectively 38% and 72% of cells) (Fig. 8C d and e). Incubation of Caco-2 cells with TCDD and endosulfan also caused an increase in Lysotracker Red staining (results not shown), indicating an important increase in lysosomal vacuole content. This strongly correlates with the increase in the acidic compartment detected by acridine orange staining.

To monitor the autophagic process, conversion of LC3-I in LC3-II, a protein involved in the autophagic process, was analyzed by quantification of LC3-II expression by Western Blot (Fig. 8D). The treatment of CaCo-2 cells with TCDD25 + α-E 10 clearly induced an enhancement of the autophagic fluxes towards the LC3-II as did the treatment with rapamycin (Fig. 8D). 3-MA and Bafilomycin A1 did so aswell. Even though it cannot be observed, the LC3-II accumulation certainly arises from the fact that LC3-II is rapidly degraded when cell death is very high. The histogram in Fig. 8E shows the resumption of the evolution of the mitochondrial ΔΨm versus the increase in LC3-II staining. Caco-2 cells in culture exposed to the pollutant mixture showed an increase in the conversion of LC3-I in LC3-II as usually described when autophagic fluxes increase.

Interestingly, when ERK1/2 activation (which is related to the Akt pathway) was measured in the presence of the individual pollutants or of cocktails of them, there was a clear induction of ERK1/2 (extracellular signal–regulated kinases) (Supplemental Fig. 2) which was related to TCDD but appeared to act synergistically when TCDD and α-E are used together. The most significant observation concerning ERK1/2 activation is that this is an early event since its maximum seems to be at 4 h.

### TCDD plus α-E cocktails associated events: calpain, cathepsin and caspase-8 activation

**Figure 9:**
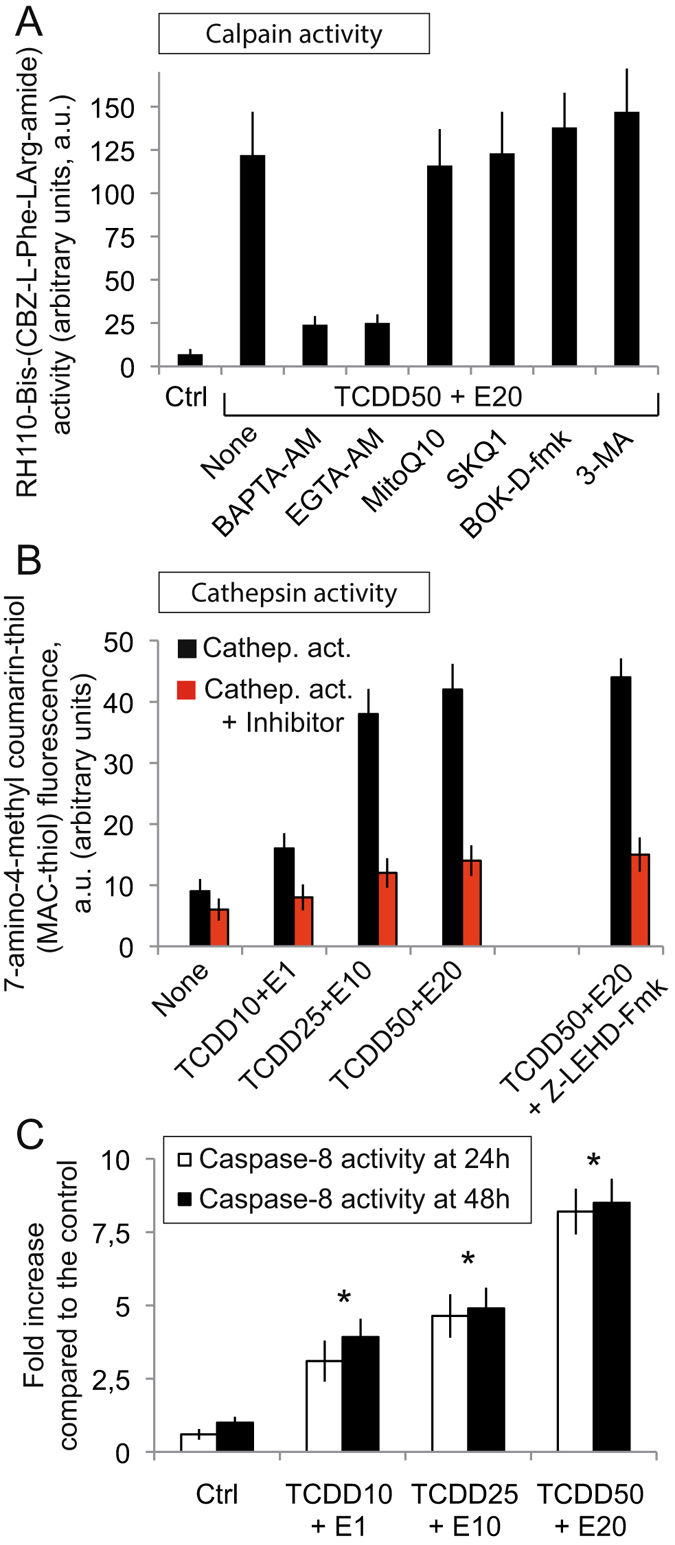
Cathepsin, calpain and caspase-8 activation by α-E, TCDD or pollutant cocktails. (**A**) Flow cytometry analysis of Caco-2 cells calpain activity following the incubation of cells with a pollutant cocktail (TCDD 50 nM + α-E 20 μM) for 24 h in the presence of calcium chelators, BAPTA-AM or EGTA-AM, mitochondrially targeted antioxidants (MitoQ10 or SKQ1), a pan caspase inhibitor BOK-D-fmk or 3 methyladenine an inhibitor of autophagy (n = 8). (**B**) Cathepsin activity in Caco-2 cells treated with a cocktail of pollutants for 48 h. A cathepsin inhibitor cocktail has been tested as well for the effects of z-LEHD, an inhibitor of caspase-9 (n = 6). (**C**) Caspase-8 activity in Caco-2 cells treated with TCDD 50 nM + α-E 20 μM has been tested at 24 h and 48 h (n = 9).

Following the ER stress response and calcium release one is likely to observe an important calpain activation that is clearly inhibitable by cell permeant calcium chelators such as BAPTA-AM [1,2-Bis(2-aminophenoxy)ethane-N,N,N′,N′-tetraacetic acid tetrakis(acetoxymethyl ester)] and EGTA-AM (Fig. 9A). The calpain activation by pollutant cocktails is not inhibitable by mitochondrially targeted ROS inhibitors like SKQ1 [Mitochondria-targeted antioxidant 10-(6-plastoquinonyl)decyltriphenyl-phosphonium] and MitoQ10 (mitochondrially targeted mitoquinone) nor by the pan-caspase inhibitor BOK-D-fmk nor influenced by 3-methyladenine (3-MA), an autophagy inhibitor (Fig. 9A). Using the same rationale, which is linked to the lysosomal destabilization that we find plausible, we measured the cathepsin activity at 48 h incubation with pollutant cocktails (Fig. 9B). This showed that an important cathepsin activity could be detected as soon as a TCDD25 + α-E10 concentration was reached, that this activity is inhibitable (by a cathepsin inhibitor cocktail), and that it is also strictly linked to the activation of the execution caspase pathway (caspase-9) since Z-LEHD-fmk primarily inhibits the cathepsin activation (Fig. 9B).

Since it has been shown in other contexts that cathepsin activation could be followed by a cleavage and activation of a caspase-8 pathway, we tested the caspase-8 activity at 24 h and 48 h induced by the different mixture of pollutants. The lower dose efficiently activates caspase-8, and the subsequent higher cocktail concentrations enhance caspase-8 activity which is almost maximalized as quickly as 24 h (Fig. 9C).

## Discussion

2,3,7,8-tetrachlorobenzo-*p*-dioxin (TCDD) is part of dioxin family. It is primarily produced through industrial processes that include incineration and manufacturing of pesticides, endosulfan among them^60^. Accordingly, TCDD is an unavoidable by-product of endosulfan synthesis. The present study was initiated to investigate the action of these pollutants in mixture at sub-lethal doses. These two products differ in their signaling pathway but were potentially act synergistically.

One of the main characteristics of these two pollutants is their lipophilicity which allows to hypothesize about their potent permeation into biological membranes. They rapidly modify membrane homeostasis and influence membrane permeability.

Initially, the incubation of Caco-2 cells with the mixtures at different concentrations induces loss of viability (Fig. 1), increases oxidative stress on lipids and proteins, increases lactate release, and induces EROD activity thus, showing important deleterious changes in cellular behavior (Fig. 2).

Clearly, these initial steps affect the endoplasmic reticulum that is undergoing a huge lumen swelling and trigger the activation of stress enzymes such as CHOP and GRP78 (Fig. 3), with a concomitant release of calcium (Fig. 2E,F). The calcium is immediately taken up by the mitochondrial calcium uniport (MCU) and provokes a mitochondrial swelling (Fig. 4A) linked to a superoxide anion, and H_2_O_2_ production (Fig. 6), as well as electron transport alteration and disruption of the ΔΨ_m_ homeostasis (Fig. 5). The increase in AMP to ATP ratio induced by pollutant cocktails is of particular importance (Fig. 5D) since this might lead to a AMP kinase activation, which is a major regulator of the PGC1-α pathway that accounts in mitochondrial homeostasis in adaptation for differential energetic demand.

In this context, treated cells are also undergoing an increase in autophagic fluxes. Since the role of lysosomes is crucial to autophagy regulation, it is important to consider that endosulfan apparently increases lysosome number and size, and can be considered as a potent stabilizer of the lysosomal membrane^61^, whereas TCDD appears to induce lysosomal fusion as well as its destabilization^62, 63^ (Fig. 7). The production of ROS by the mitochondria and the increase of lysosome size and number in the initial time period, allows the consideration of a possible induction of autophagic fluxes and may explain what many researchers have attributed an anti-apoptotic function to TCDD^27–29^.

From our point of view, low concentration mixtures of TCDD + α-E are apparently favoring cellular proliferation in a context where the initial consequences rely upon autophagic flux increases (Fig. 8). Whatever the method use: Study of the cell proliferation with the impedancemetry technique, stain of the acidic compartment with AO, or the correlation of it with the expression of the autophagic marker LC3-II (Fig. 8D–F) the increase in the autophagic processes end when the cell death events reach a certain threshold (Fig. 8C and D). From this point of view it is likely that the use of 3-MA, an autophagy inhibitor, increases the percentage of dead cells (Fig. 8E). In addition, it results in the activation of the ERK1/2 phosphorylation pathway since the Akt and ERK1/2 pathway has been demonstrated to be related to the proliferative and anti-apoptotic effect of TCDD in lung tumors^29^. With the ERK1/2 stimulated very early in the exposure (Supplemental Fig. 2) and the phosphorylation decreasing later on at 24 h and 48 h, one may speculate that this is effectively linked to cell death inhibition which is concurrent with the autophagy induction by TCDD and certainly by the pollutant cocktails.

The caspase activation which follows (Fig. 9) slowly leads the cells to pass a certain threshold above which they undergo rapid death processes. Within these complex networks of events, the partial disruption of lysosome membrane permeability is linked by cathepsin activation and follows calcium-induced calpain activation (Fig. 9). It continues on a clear activation of caspase-8 and its subsequent counterparts caspase-3/-9 (Fig. 9C).

**Figure 10:**
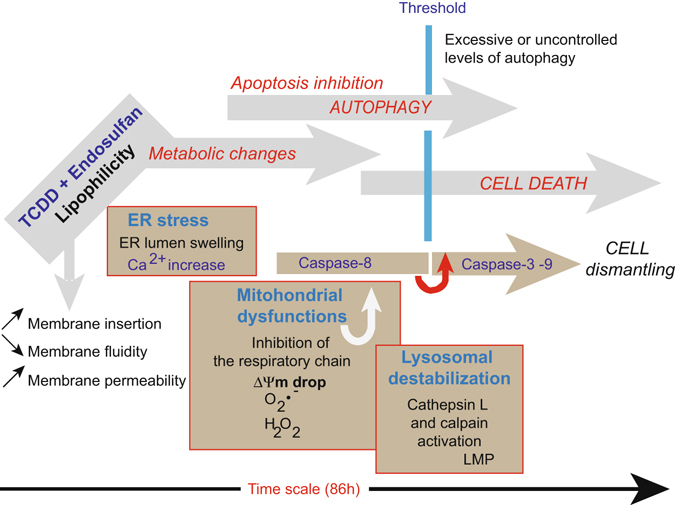
Schematic representation of the various events involved in the TCDD + endosulfan signalization. TCDD and endosulfan which are very lipophilic substances penetrate the cells and interact with all the membrane compartments. They provoke an ER stress and lysosomal membrane destabilization. Calcium release from the ER enter the mitochondria and alter the electron transport chain, resulting in a ΔΨm drop and superoxide generation. Altered mitochondria are taken up by mitophagy. The lysosomal pathway also converges towards mitochondria since calpain and cathepsin are susceptible of Caspase-8 activation and Bid cleavage. Huge autophagic processes are induced in parallel to cell death events until Beclin-1 is cleaved and death dominates. An inhibition of autophagy (by Bafilomycin A1) leads to a more pronounced cell death.

As a partial conclusion, mixtures of TCDD and endosulfan act synergistically to induce harmful effects in human cells. One of the main characteristics of these two pollutants is their lipophilicity that allow one to hypothesize about their potent permeation into biological membranes (Fig. 10). Cell death occurring after a long autophagic sequence is characterized by a perinuclear space with lumen, stress fiber accumulation as well as an accumulation of autophagosomes and loss of plasma membrane permeability.

Exogenous ligands of the aryl hydrocarbon receptor (AhR) such as 2,3,7,8-tetrachlorodibenzo-p-dioxin (TCDD) and related substances are potent toxic pollutants that still persist in the environment. Combined effects of low concentrations of TCDD and endosulfan on autophagy and cell death differed from that of individual compounds. The results herein, clearly indicate that synergistic effects exerted by low doses of mixtures must be taken into consideration when studying new formulation to be released in the market or environmental risk assessment. It is likely that the increase in autophagic fluxes constitute a cardinal feature of the propensity for a mixture to exacerbate cancer progression *in vivo*.

## Materials and Methods

### Chemicals

TCDD was prepared from a 0.155 mM stock solution (Promochem, Molsheim, France) and used at concentrations of 10, 25, 50 nM together with α-endosulfan (α-E) (Promochem, Molsheim, France) used at 1, 10 and 20 µM. The mixtures used in this work are TCDD 10 nM + α-E 1 µM, TCDD 20 nM + α-E 10 µM and TCDD 50 nM + α-E 20 µM.

### Cells

The Caco-2 cell line is a continuous line of heterogeneous human epithelial colorectal adenocarcinoma cells, developed by the Sloan-Kettering Institute for Cancer Research. Caco-2 cells were seeded at 7.5 × 10^5^ cells/cm^2^ in 6-well plates or in 25 cm^2^ flasks and cultured in Dulbecco’s Modified Eagle’s essential Medium (DMEM Glutamax-I) containing 4,5 g/L glucose and supplemented with 20% fetal calf serum (FCS), 1% penicillin-streptomycin, HEPES NaOH 1 mM, Na-pyruvate 1 mM and 1% non-essential amino acids (MEM, GIBCO). Cultures were maintained in a 5% CO_2_/95% air atmosphere.

### Mitochondrial membrane potential, ROS production and calcium content

Caco-2 cells were treated with different mixtures. These include TCDD 10 nM + 1 µM α-E, TCDD 25 nM + 10 µM α-E or 50 nM TCDD + 20 µM α-E for 48 hours unless stated otherwise. After treatment, cells were trypsinized, washed and resuspended together with their supernatant in the DMEM Glutamax-I culture medium at 10% FSC. The following probes were added to cell samples and incubated for 15 minutes at 37 °C. For mitochondrial membrane potential determination, 3,3′-dihexyloxacarbocyanine iodide (DiOC_6_(3), Molecular probes) was used at 20 nM final concentration (stock solution 10 μM). The uncoupling agent carbonyl cyanide m-chlorophenylhydrazone (mClCCCP, 1 mM stock solution, 15 min, 37 °C) was added at 10 μM before dye addition and used as a control of ΔΨm drop; for ROS production. 2′,7′-dichlorodihydrofluorescein diacetate (H_2_DCFH-DA, Dichloro-dihydro-fluorescein diacetate, Molecular probes) was used at 5 µM final concentration for H_2_O_2_ measurements (stock solution at 1 mM) and MitoSOX (Molecular probe) was used at 5 µM final concentration for superoxide anion (Stock solution at 1 mM). H_2_O_2_ (5 μM, 30 min) was used as a positive control for the H_2_DCF-DA measurements and paraquat 50 μM was used for superoxide anions generation. For cytosolic calcium determination, Fluo4-AM (1 mM stock solution, Molecular probes) was used at 5 µM final concentration, as previously described^58, 64^. Ca^2+^ titration of Fluo4-AM was performed by reciprocal dilution of Ca^2+^-free and Ca^2+^-saturated buffers prepared by using a Ca^2+^ kit buffer (Invitrogen) on cells poisoned with DPBS supplemented with mClCCCP (10 μM) and ionomycin (5 µM)^65^. Most of the time a double staining was realized to simultaneously assay the cellular viability, with propidium iodide (PI) at 1 mg.ml^−1^ final concentration for DiOC_6_(3), H_2_DCFH-DA, YO-PRO-1 and Fluo4-AM or, with TO-PRO-3 iodide at 1 mg.ml^−1^ final concentration when using MitoSOX staining. A supplemental double staining was used for the distinction between viable, apoptotic and necrotic cells with YO-PRO-1/PI in parallel with an Annexin-V/PI staining performed with the Annexin-V FITC (Immunotech, Beckman-Coulter) at 1 mg.ml^−1^ final concentration in presence of calcium in order to detect the aberrant exposure of phophatidyl-serine residues at the outer surface of the plasma membrane (Fig. 1B, *rational in a red box). For the cell sorting (Fig. 1A), the cells are selected on the PI staining, the sorted cells were either PI^low^ (control, viable cells) PI^intermediate^ or PI^high^ (sorted cells). They were reanalysed after staining with annexin-V-FITC in a calcium buffer plus TO-PRO-3 and reanalyzed instantaneously. To test the effects of ROS scavengers, N-acetylcystein (NAC; 1 mM), MitoQ_10_ (5 μM) and SkQ1 (500 nM) Caco-2 cells were preincubated 3 h with the antioxidants before treatments. Cellular viability (PI) together with ΔΨm [DiOC_6_(3)] was measured.

### Analysis of cellular respiration with the seahorse XF4 system

The oxygen consumption rates were measured using an XF24 high sensitivity respirometer as described previously^66^. In short, 50,000 cells (with or without pollutants treatment) were cultured in DMEM glutamax for 16 h and the medium was replaced with XF assay medium (low buffered bicarbonate-free DMEM at pH 7.4) before measurement. The cells were incubated at 37 °C for 30 min to allow media temperature and pH to stabilize before the first rate measurement. Oligomycin (2 μg/ml), mClCCP (carbonyl cyanide m-chlorophenylhydrazone, a mitochondrial uncoupler, 10 μM), or the complex I inhibitor rotenone (1 μM) were injected through the cartridge, and oxygen consumption rates were measured under basal conditions as well as after sequential injections of oligomycin, 2,4-dinitrophenol, and rotenone after calibration Measurement cycles of 1-min sample mixing, 2 min waiting, and 3 min measurement of oxygen consumption rate (OCR) were carried out.

### Mitochondrial respiratory complex activities

Mitochondrial respiratory complex activities were measured as previously described^67^. The complex I activity was recorded as decreasing absorption at 340 nm (NADH oxidation) of reaction mixtures containing 200 μM NADH and 100 μM ubiquinone-1 in the presence of 1.5 mM KCN, and the rotenone-sensitive activity was calculated. The activity of cytochrome *c* oxidase was measured spectrophotometrically by following the oxidation of ferrocytochrome *c* at 550 nm. The standard assay mixture contained the following: 0.25 M sucrose, 50 mM Tris-HCl, pH 8.0, 0.2 mM potassium EDTA, and mitochondria (10 μg of protein/ml).

### NAD(P)H determination by flow cytometry

NAD(P)H fluorescence was elicited with a multiline ultraviolet light set at 400 mW on a FACS Vantage. Changes in the autofluorescence of normal and apoptotic cells were recorded as previously described by Gendron *et al*.^64^.

### Determination of lipid peroxidation, protein carbonylation, lactate production and EROD activity (as reporter of CYP1 A1, CYP1A2 and CYP1B1)

We used a lipid peroxidation assay kit (Abcam) to detect malonaldehyde (MDA) present in samples. The free MDA generated during lipid peroxidation refers to the oxidative degradation of lipids reacts with Thiobarbituric Acid (TBA) to generate a MDA-TBA adduct. The absorbance of TBA-MDA adduct was measured at 532 nm and this kit detects levels as low as 1 nmol/well. For the calculation, we determined the MDA concentration in standards and samples from their absorbance as described in the protocol of Lipid peroxidation assay kit from Abcam (ab118970). Protein carbonylation has been assayed using the Cayman’s Protein Carbonyl Fluorometric Assay Kit in Caco-2 cell lysates. Cytotoxicity was assessed by measuring the release of lactate dehydrogenase (LDH) into the medium using the CytoTox96 LDH assay kit (Promega, France). Culture supernatant (50 mL) was incubatedwith an equal volume of LDH substrate solution in dark conditions for 30 minutes. The reaction was stopped with 50 mL of 1 M acetic acid, and the absorbance was determined at 492 nm. The EROD activity in cultured cells was measured by the fluorescence of resorufin generated by the conversion of ethoxyresorufin by CYP1A1, CYP1A2 and CYP1B1 using a fluorescence plate reader^68^. For lipid peroxidation, protein carbonylation, lactate production and EROD activity, all experiments have been carried at least 10 times unless noticed).

### Western blots

Cells were lysed in modified Laemmli’s buffer [60 mM Tris (pH 6.8), 10% glycerol (vol/vol), 2% (wt/vol) SDS and Bromophenol Blue, without 2-mercaptoethanol] by sonication for 30 s on ice and were then centrifuged at 3000 g for 5 min. The supernatants were boiled for 5 min at 100 °C and frozen at −80 °C. Protein concentration was determined by the micro-BCA protein assay (Pierce, Rockford, IL, USA). Cell lysates (20 μg per lane) were resolved by SDS-PAGE (7.5% or 15% (wt/vol) polyacrylamide). Proteins were then electroblotted onto 0.45 μm pore-size nitrocellulose filters, and the filters were blocked by incubation with 5% (wt/vol) non-fat milk in PBS containing 0.1% Tween-20 for 1 h and the filters were blocked by incubation with 5% (wt/vol) non-fat milk in PBS containing 0.1% Tween-20 for 1 h. The filters were then incubated for 1 h at room temperature to determine LC3 cleavage, with rabbit anti-LC3b (ref. L7543 from Sigma-Aldrich) at 1:1000 and detected with anti-rabbit, RPN2124 from GE Healthcare (Vélizy-Villacoublay, France) at 1:30 000. Blots were washed three times for 10 min with 0.2% Tween 20 in PBS, then incubated for 1 h with peroxidase-labeled anti-mouse or anti-rabbit immunoglobulins (at 1:5000 dilution). Blots were developed using an enhanced chemiluminescence detection system (ECL2; Amersham, UK).

### Acridine orange staining of the acidic cellular compartment and LC3-II determination by flow cytometry

Caco-2 cells, control or treated with various concentrations pollutants cocktails for different times, were incubated with 2 μM AO for 30 min at 37 °C. The samples were analyzed by flow cytometry. AO preferentially accumulates in acidic vesicles and generates red fluorescence (aggregates) upon excitation at 488 nm whereas when intercalated at the DNA level, generates a green fluorescence. These measurements required completion by other classical methods to determine autophagy, i.e. electron microscopy and LC3 cleavage. In our case, we analyzed the presence of LC3-II by flow cytometry determination by using Anti-LC3B polyclonal antibody (0.25 mg; cat. no. L10382, Invitrogen) or rabbit immunoglobulin (0.25 mg; cat. no. I5006, Sigma Chemicals) was used as an isotype control and incubated for 30 min at 37 °C. Cells were then washed in PBS buffer and labeled with 125 μg of secondary fluorescent conjugate Alexa Fluo-488 goat anti-rabbit IgG (cat. no. A11008, Invitrogen) for 30 min at 37 °C. Following this, cells were then washed in PBS buffer and resuspended in 400 μl of PBS in the presence of propidium iodide (1 μg/μl) and then analyzed in flow cytometry.

### Caspase-3, -8 and -9 activity assays for flow cytometry

Controls of treated Caco-2 cells were plated in 6-well plates at a density of 2.10^6^ cells/well and incubated for various periods of time (0 to 48 h) with the appropriate mixture of pollutants and then harvested. The samples were then washed, resuspended in 50 μl of 10 μM substrate solutions (PhiPhiLuxG1D2 for caspase-3, CaspasLux8 for caspase-8 and CaspasLux9 for caspase-9), and incubated at 37 °C for 1 h according to the assay manufacturer’s (Oncoimmun, USA) instructions. The cells were then washed again with PBS and were analyzed by flow cytometry.

### Analysis of cathepsin and calpain activities by flow cytometry

Cathepsin and calpain activity in live cells was determined with the use of specific substrates as previously described in ref. 69. More detailed explanations are given in supplementary Materials and methods for cathepsins and calpain, respectively.

### AMP/ADP/ATP determination

Adenine nucleotides were separated by HPLC on a C18 column (Polaris 5C18-A, S250*4.6 Repl, Varian, France). The injection volume was 30 μL. The Flow rate was 1 ml/min, and the cartridge was kept at 30 °C in a column oven. The mobile phase was 28 mM pyrophosphate buffer, pH 5.75. These compounds were detected on a spectrophotometer at a wavelength of 254 nm (L4200 UV Detector, Merck, USA). The retention times of ATP, ADP and AMP were 3, 5 and 9 min, respectively. Chromatograms were integrated with STAR software v.5 (Varian, France).

### Transmission electron microscopy

Caco-2 cell pellets were fixed by incubation with 2.5% glutaraldehyde in 0.1 M cacodylate buffer (pH 7.4) for at least 30 min at 4 **°**C. The fixed specimens were thoroughly washed in 0.1 M cacodylate buffer and then post-fixed by incubation in 1% osmium tetroxide in the same buffer for 1 h at room temperature, bulk-stained with 2% uranyl acetate in distilled water for 15 min, dehydrated in a graded series of acetonitrile concentrations, and embedded in Epon resin. Ultrathin sections (80**–**100 nm thick) mounted on 150-mesh grids were stained with 2% uranyl acetate solution and Reynolds lead citrate. When needed the experiments are done 5 times and 10 images realized for each conditions in order to access statistical values.

### Statistics

Stastistical analysis has been carried out with the Kruskal-Wallis test (*p < 0.05; **p < 0.01; ***p < 0.001). Data are expressed as mean ± SD with the number of experiments cited as n.

## Electronic supplementary material

Supplemetary information
