# Supplementary material for: Synergistic cellular effects including mitochondrial destabilization, autophagy and apoptosis following low-level exposure to a mixture of lipophilic persistent organic pollutants
Source: Sci Rep. 2017 Jul 5;7:4728. doi: 10.1038/s41598-017-04654-0 (PMC5498599; doi:10.1038/s41598-017-04654-0)

Synergistic cellular effects including mitochondrial destabilization, autophagy and apoptosis following low-level exposure to a mixture of lipophilic persistent organic pollutants

Nathan E. Rainey[1,2], Ana Saric[1,3], Alexandre Leberre[1], Etienne Dewailly[4], Christian Slomianny[4], Guillaume Vial[5], Harold I. Zeliger [6], and Patrice X. Petit[1]

Supplementary informations

Supplementary Figures legends

Supplementatry Figure 1 – TCDD-induced lysosomal exocytosis. Caco-2 cells were treated with DMSO + Nonane (DMSO + N, as a control), TCDD 50 nM or ionomycin 3 mM for the indicated period of time. Cells where also treated with various cocktails of pollutants, TCDD + Endosulfan as indicated. The supernatants of the cell cultures were assayed for the release of lysosomal β-hexaminidase, and the data were expressed as a percentage of the total cellular content of the enzyme (mean ± SEM, n = 10).

Supplementary Figure 2 - ERK1/2 activation as analysed flow cytometry when Caco$_2$ cells are treatd by individual pollutants or pollutant cocktail. The antibody used at 1/1000 is the anti-ERK1/2 (pT202/pY204)-488 (clone: 20A RUO from BD Biosciences) following manufacturer instructions. The 20A monoclonal antibody recognizes the phosphorylated threonine 202 and tyrosine 204 (pT202/pY204) of human ERK1 and pT184/pY186 of human ERK2. Alexa Fluor® 488 fluorochrome emission is collected with a FL1 Band Pass 530 ± 40 nm on the FACSCalibur 4C.

Supplementary Material and Methods (more detailed)

Supplementary M&M 1. Determination of EROD activity (as reporter of CYP1 A1), protein carbonylation and lipid peroxidation.

The Cayman's Protein Carbonyl Fluorometric Assay Kit (Kit 700490) provides a reliable and sensitive method for determining protein carbonyl concentration in plasma, serum, cell lysate, and tissue homogenate samples. The assay relies on the 1:1 binding of a fluorophore to the protein carbonyl contained in a cell lysate. Once bound, excess fluorophore is washed away. Any remaining fluorescence is directly proportional to

protein carbonyl concentration (Kit 700490). The degree of lipid peroxidation was determined in the form of malondialdehyde (MDA) as a thiobarbituric acid-reactive substance (TBARS). In brief, an aliquot of 50 ml of 3–5 X10^6 cells was pipetted into a test tube containing 0.6 ml of 0.44 M phosphoric acid. After mixing with 0.2 ml of a 42 mM thiobarbituric acid solution, it was then placed in a 95°C dry bath for 1 h. The samples were then cooled and neutralized with 1 N NaOH in methanol before the high performance liquid chromatography (HPLC) analysis. An aliquot of 20 ml of supernatant was injected into a C18 column (4.6 X 250 mm, with a particle size of 5 mm) using a Jasco PU-980 pump (Easton, MD) with a solvent system composed of methanol and 50 mM phosphate buffer (pH 6.8; 4:6, v/v) at a flow rate of 1 ml/min. The eluent was monitored with a fluorescence detector with the excitation wavelength at 525 nm and emission wavelength at 550 nm.

Supplementary M&M 2. Analysis of cathepsin activity by flow cytometry. Cathepsin activity in live cells was determined with the use of AMC substrates as previously described in [74]. Stock solutions of substrates and inhibitors coktail in DMSO were stored at - 80°C [(Z-Arg)2 Rh110, 2HCl], E64d, pepstatin or selective inhibitors for cathepsins L and S where Z-Phe-Phe-CHN2 and Z-Val-Val-Nle-CHN2, respectively. Percent inhibition was calculated as [1-(fluorescence units with inhibitor/ fluorescence units without [74]inhibitor)] X 100%.

Supplementary M&M 3. Analysis of calpain activity by flow cytometry. 7-Amino-4-chloromethyl coumarin, t-BOC-leucine-methionine amide (BOC-LM-CMAC) was purchased from Invitrogen; it is a calpain substrate which is well retained in live cells and for which the substrate and the product have different spectral properties. The non-fluorescent-BOC-L-leucyl-methionine amide diffuses freely into cells but then becomes unable to cross membranes after being conjugated to a thiol. Cleavage of t-BOC-thiol by calpain results in the release of fluorescent 7-amino-4-methylcoumarin-thiol (MAC-thiol). The cells were incubated with BOC-LM-CMAC (10 μM) for 30 minutes at 37°C, trypsinized and the fluorescence at 405 ± 30 nm of aliquots of 10,000 cells analyzed with a FACS Aria III with excitation at 367 nm. PD 150606 (Enzo ref. ALX-270-234-M005), a cell-permeable non-competitive inhibitor of calpains 1 and 2 was used as calpain activity inhibitor at 500 nM).

Supplementary M&M 4. Flow cytometry analysis of CHOP and GRP78 activities. Caco-2 cells were treated with different cocktails of pollutants or pollutants alone for 6 h

or 12 h and tested for GRP78 and CHOP activity. Cells were grown in 6 wells plates, trypsinized and then fixed by 4% paraformaldehyde at 4°C for 40 min and rinsed several times with PBS. Nonspecific binding sites were blocked for 2 h at room temperature with 5% normal SVF (Gibco, ThermoFisher Scientific, France) in 0.1% Triton X-100-PBS. Caco-2 cells were incubated overnight at 4°C with primary antibodies (1:100 dilutions with blocking buffer) for GRP78 (Cell Signaling, MA, USA) or CHOP (Santa Cruz, CA, USA). Cells were then incubated with a mixture of fluorescein isothiocyanate- and tetramethyl rhodamine isothiocyanate-conjugated secondary antibodies (BD Biosciences, France) for 2 h at 4°C. Cells were analysed by flow cytometry using the green (FL1 = 525 ± 30 nM) or the red (FL-2 = 585 ± 42 nm) channels. Each experiment was repeated a minimum of four times in duplicates.

Supplementary Figure 1

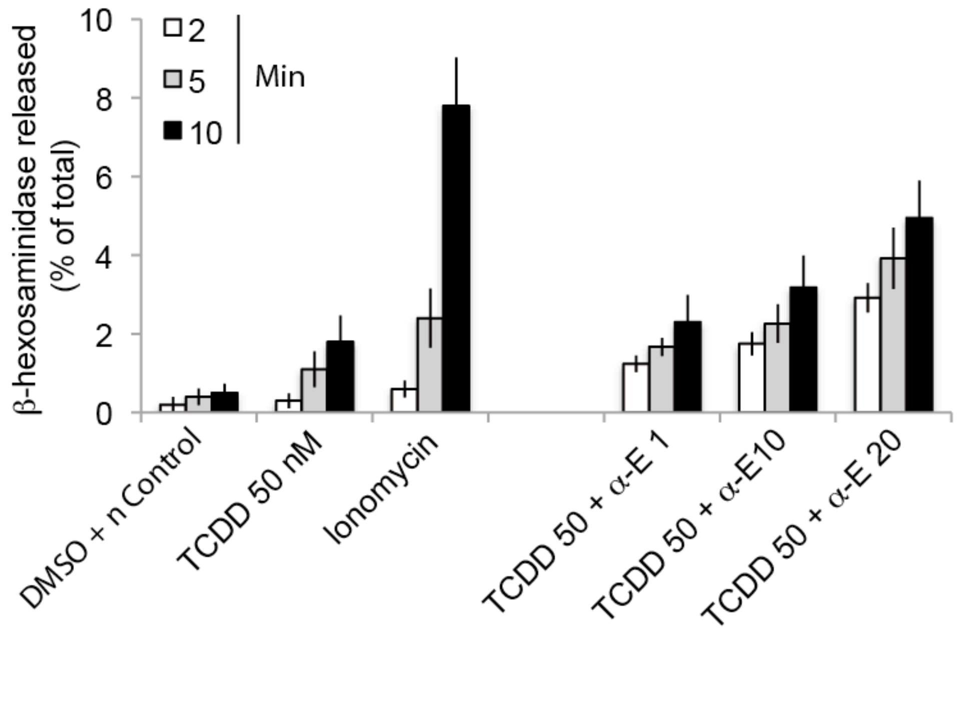

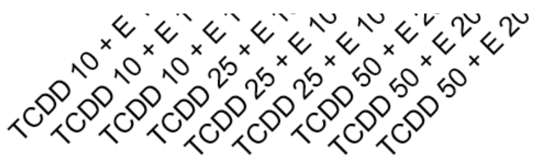

Supplementary Figure 2

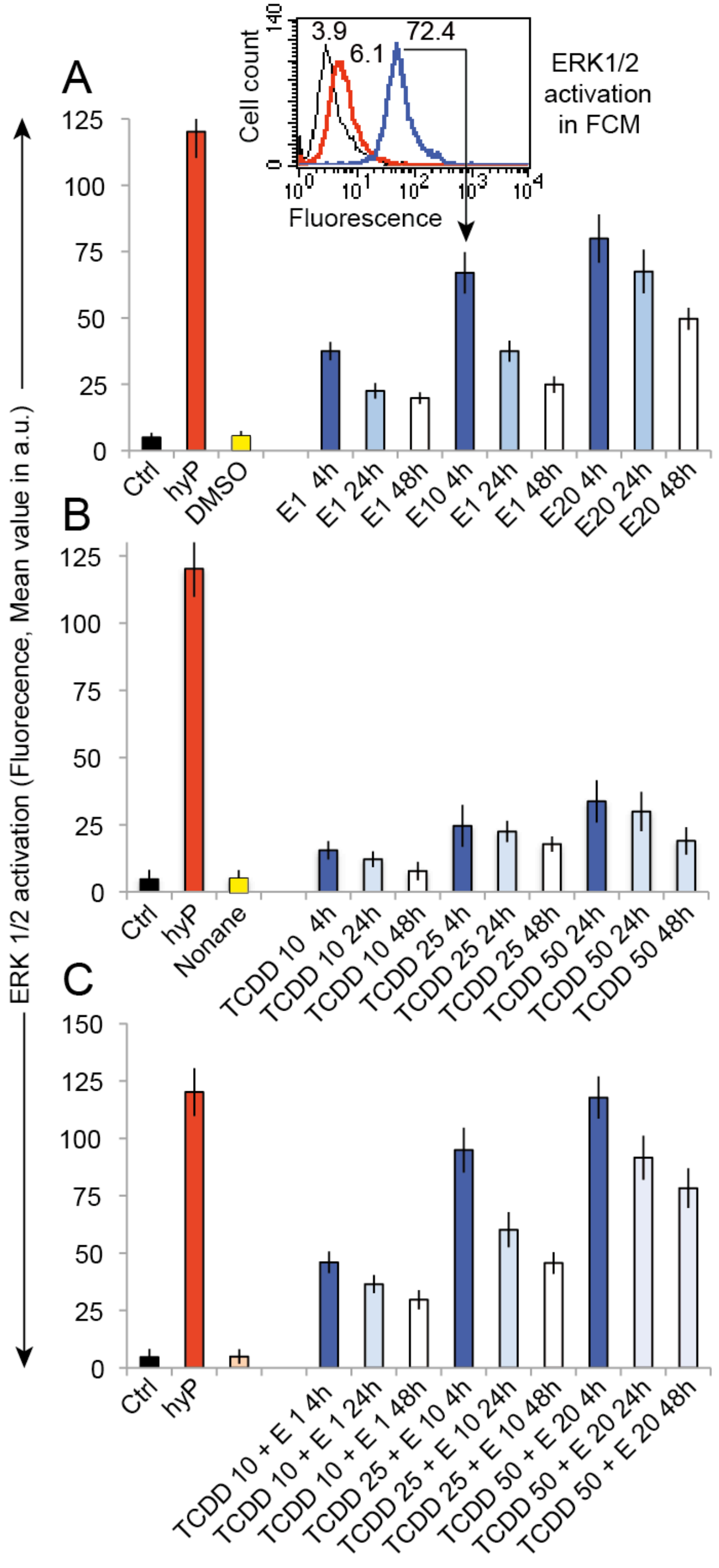

Supplement: Supplementary file 1 — Supplemetary information [file 41598_2017_4654_MOESM1_ESM.pdf]
